# Asymptomatic Chronic Large Pericardial Effusions: To Drain or to Observe?

**DOI:** 10.3390/jcm13133887

**Published:** 2024-07-02

**Authors:** Emilia Lazarou, Charalambos Vlachopoulos, Alexios Antonopoulos, Massimo Imazio, Antonio Brucato, Costas Tsioufis, George Lazaros

**Affiliations:** 1 First Cardiology Department, School of Medicine, Hippokration General Hospital, National and Kapodistrian University of Athens, Vas. Sofias 114, 11527 Athens, Greece; emilazarou@gmail.com (E.L.); cvlachop@otenet.gr (C.V.); antonopoulosal@yahoo.gr (A.A.); ktsioufis@gmail.com (C.T.); 2 Department of Medicine (DMED), University of Udine, 33100 Udine, Italy; massimo.imazio@uniud.it; 3 Cardiothoracic Department, University Hospital Santa Maria della Misericordia, 33100 Udine, Italy; 4 Department of Biomedical and Clinical Sciences, Luigi Sacco Hospital, University of Milan, Milan, Italy; antonio.brucato@asst-fbf-sacco.it

**Keywords:** pericardial effusion, pericardial drainage, etiology, management, outcome

## Abstract

Pericardial effusions, especially large ones, have traditionally been regarded with concern by clinicians due to the sometimes unpredictable development of life-threatening cardiac tamponade. In the European Society of Cardiology Guidelines on pericardial diseases, the simplified algorithm for pericardial effusion triage and management recommends pericardial drainage in cases of cardiac tamponade and/or suspicion of bacterial or neoplastic etiology. In the presence of acute pericarditis, empiric anti-inflammatory treatment should be given, while when a specific indication known to be associated with pericardial effusion is found, then treatment of the underlying cause is indicated. Notably, the most challenging subgroup of patients includes those with large, asymptomatic, C-reactive-protein-negative, idiopathic effusions. In the latter subjects, pericardial drainage is proposed in cases of chronic effusions (lasting more than three months). However, this recommendation is based on scant data stemming from small-sized non-randomized studies. Nevertheless, recent evidence in a larger cohort of patients pointed out that a watchful waiting strategy is a safe option in terms of complication-free survival. This review summarizes the contemporary evidence on this challenging topic and provides recommendations for tailoring individual patient treatments.

## 1. Introduction

Pericardial syndromes have received particular attention in recent years, especially during the COVID-19 pandemic, since it is well known that they may complicate either SARS-CoV-2 infection or, less frequently, vaccination against COVID-19 mainly with the adoption of the mRNA vaccine platform [1,2,3,4]. Notably, although the management of the most common pericardial syndrome, namely acute pericarditis, is well established following extensive research on this condition, evidence on the treatment of other pericardial syndromes, including pericardial effusions, is less solid and, in many instances, the relevant data are quite controversial [5,6,7].

Specific pericardial effusion, in the setting of acute pericarditis, should be treated with an empiric anti-inflammatory treatment according to the recommendations of the 2015 European Society of Cardiology Guidelines for the diagnosis and management of pericardial diseases [6]. However, significant controversy surrounds pericardial effusions, especially chronic ones (i.e., lasting more than 3 months), in the absence of evidence of ongoing pericardial inflammation, as depicted by C-reactive protein elevation or by imaging (mainly pericardial edema and late gadolinium enhancement by cardiac magnetic resonance imaging) [5,6,7]. 

For several years, the latter entity has been regarded as an alarming condition potentially heralding cardiac tamponade [7]. Thus, pericardial drainage according to the Guidelines is ‘to be considered’ in chronic idiopathic CRP-negative pericardial effusions [6]. However, the supporting data on this recommendation were based on weak evidence stemming from small observational studies [7]. Nevertheless, recent larger-scale studies were quite reassuring about this condition, especially in asymptomatic individuals, and suggest a less aggressive approach with a watchful waiting strategy [8,9,10].

In this review, we have addressed the problematic topic of pericardial effusions, giving priority to recent evidence and gaps in knowledge and individualized treatment according to the specific clinical scenario. 

## 2. Outlines of Pericardial Anatomy and Physiology

The normal pericardium is a fibrous-serous double-walled structure surrounding the heart and the root of the great vessels [11,12]. The inner sac is called the serous pericardium, and the outer is the fibrous pericardium. The serous pericardium is also composed of two layers, one internal (visceral pericardium or epicardium) adjacent to epicardial fat and the heart muscle and the other external (parietal pericardium) in contact with fibrous pericardium [12]. Parietal pericardium reflects at the level of the great vessels route and continues with the visceral layer [11,12]. The two layers of the serous pericardium form the pericardial cavity, which is a virtual spacey containing 10–50 mL of fluid. Normal pericardial thickness varies between 0.7 and 3 mm depending on the imaging modality adopted for measurement [5].

The amount of pericardial fluid depends on the balance of hydrostatic and colloid osmotic pressures between the visceral pericardium and pericardial cavity. In normal conditions, there is a net driving pressure of ~2–10 mmHg from the visceral pericardium to the pericardial cavity, whereas the lymphatic vessels are mainly in charge of removing excess fluid [12]. The pericardial effusion volume may increase in several pathological conditions such as fluid overproduction in cases of pericardial inflammation or post-traumatic intrapericardial hemorrhage, increased hydrostatic pressure due to heart failure, and/or pulmonary hypertension and decreased colloid osmotic pressure in disorders causing hypoalbuminemia [5]. Finally, an impairment in local lymphatic circulation, for instance, due to malignant lymphatics invasion may alternatively account for pathologic fluid accumulation (pericardial effusion) [11,12].

Several functions have been attributed to the pericardium, including the mitigation of the friction of the heart muscle with the surrounding tissues, fixation of the heart in the mediastinum, which allows for a more efficient cardiac output, the prevention of the extensive distension of heart chambers in the presence of volume overload, preventing cardiac valves regurgitation, and finally protection against the propagation of infections from nearby structures [5]. Nevertheless, the absence of pericardium is compatible with normal life expectancy as it is testified by cases with congenital partial or complete absence of the pericardium [13,14].

The pericardium as an elastic structure has a limit of distensibility. Thus, fluid overproduction in a first instance is counterbalanced by pericardial distension [15]. However, there is a critical point where the overstretched pericardium cannot accumulate a further amount of fluid. In the latter cases, additional fluid accumulation is performed at the expense of heart chambers’ compression [15]. Right heart chambers are mostly affected in similar situations due to their smaller thickness [5,15,16]. However, in certain clinical scenarios such as tricuspid regurgitation and pulmonary hypertension, a larger volume of pericardial effusion is required for right chambers’ collapse and, accordingly, cardiac tamponade due to the elevation of diastolic right ventricular diastolic pressures in these settings. To summarize, the series of events observed in overt cardiac tamponade include right heart chambers collapse, reduced left ventricular preload, decreased stroke volume, and low cardiac output syndrome [15]. Cardiac tamponade is an emergent life-threatening condition where emergent pericardial drainage is a life-saving procedure [6]. 

## 3. Epidemiology and Causes

Pericardial effusion is a common pericardial syndrome with an estimated incidence of 3% and a prevalence of 5.7–9% [5,17]. Most of the epidemiological data have been obtained from the Western world and mainly from specialized referral centers, and thus possibly subjected to referral bias [6]. Relevant data from low-resource areas where tuberculosis is the main cause of pericardial syndromes overall are very scant [18,19]. The contemporary International Classification of Diseases (ICDs) coding system raises additional concerns regarding the true frequency of pericardial effusions [20]. This coding tool depicts low reliability regarding the correct categorization and may accordingly lead to diagnostic misclassifications. In this context, pericardial effusions are often misreported as acute pericarditis [20].

Similarly, data on the eventual differences according to sex and age are scarce and limited to the presence of pericardial effusions in acute pericarditis where older patients present more often with pericardial effusions compared to younger individuals without any difference related to sex [5]. In pericardial effusions without evidence of concomitant pericardial inflammation, no differences based on sex have emerged either [9].

From an etiological point of view, pericardial effusions may have infectious and non-infectious causes. Infections etiologies (mainly viral infections) account for 15–30% of cases and are characterized by the elevation of inflammatory markers such as C-reactive protein, the erythrocyte sedimentation rate, and white blood cells [5]. Imaging, especially cardiac magnetic resonance imaging, reveals ongoing pericardial inflammation through pericardial edema in T2 sequences and pericardial late gadolinium enhancement [21,22,23,24]. 

In developing countries, mycobacterium tuberculosis accounts for at least 70% of acute pericarditis cases [18]. Notably, in the setting of acute pericarditis, pericardial effusions (newly appearing or increasing in size) are included in the four main diagnostic criteria for acute pericarditis [6]. Pericardial effusion in acute pericarditis is observed in ~60% of causes and is mostly mild (~80% of cases) [6,25,26]. 

On the other hand, non-infectious causes of pericardial effusions include cancer (10–25%), iatrogenic causes (15–20%), and autoimmune/auto-inflammatory diseases (5–15%), while tuberculosis is the most common cause (>60%) in endemic areas [6,19,27,28]. Despite extensive diagnostic work-up, unfortunately half of the cases in the Western world are finally classified as idiopathic, while in the subgroup of chronic, large, asymptomatic, idiopathic, non-inflammatory effusions, the possibility of unveiling a specific etiology is as low as 7% [6,9,29].

It is worth reporting that two additional causes of pericardial effusions emerged quite recently. The first is SARS-CoV-2 infection, which emerged as an important cause of pericardial effusion during the COVID-19 pandemic [4,30]. Notably, pericardial effusions, inflammatory or not, may appear either in the setting of coronavirus infection or less frequently upon vaccination against COVID-19, especially with mRNA vaccine platforms [4,30]. A reasonable (although quite arbitrary) timeslot required to characterize a newly detected effusion as SARS-CoV-2 or vaccine-related is 4–6 weeks [31].

The second challenging cause is anticancer therapy in the era of the widespread use of immune checkpoint inhibitors. Pericardial effusion in cancer patients may be caused by the neoplastic invasion of the pericardium, irradiation therapy, chemotherapy, and immunosuppression predisposing to infections, including acute pericarditis [6,32,33]. Defining the etiology is sometimes difficult, and pericardiocentesis with pericardial fluid analysis, if technically feasible, is of paramount importance for etiological search and prognostic purposes [6]. Multimodality imaging also provides valuable information on the etiology of effusion in this setting [33]. In the specific context of immune checkpoint inhibitors, the detection of moderate or large pericardial effusion with or without pericarditis is a reason for the discontinuation of the medication and the administration of steroids plus colchicine in the presence of documented pericardial inflammation, according to the 2022 European Society of Cardiology Guidelines on Cardio-Oncology [32,33]. Restarting the drugs should be considered in a multidisciplinary setting in an individualized manner [33].

## 4. Classification Diagnosis and Symptoms

Pericardial effusions are classified according to their duration, size, distribution, hemodynamic consequences, and fluid composition (Table 1) [34,35].

In brief, the size of the composition is obtained in a semiquantitative manner by echocardiography [34]. The maximal echo-free space measured at the end-diastole is employed for the classification of the effusions in small (up to 10 mm, which corresponds to approximately 100 mL of fluid), moderate (between 10 and 20 mm, 100–500 mL), and large (greater than 20 mm, >500 mL) effusions [6,34]. However, all pericardial spaces should be separately assessed and measured for reasons of completeness [5]. Second level imaging with computed tomography and cardiac magnetic resonance imaging as well as transesophageal echocardiography allow for a quantitative assessment of fluid volume and may provide valuable information on pericardial fluid composition [6].

Small effusions, for reasons of gravity, first appear in the left atrioventricular groove in the parasternal long-axis view and in the area adjacent to the right atrium in the apical four-chamber view [34]. As the fluid accumulates further, the effusion becomes circumferential [34].

The clinical presentation covers a wide spectrum of manifestations ranging from a completely asymptomatic patient to a patient with severe symptoms such as dyspnea, fatigue, palpitations, or even circulatory collapse due to cardiac tamponade [5]. The subset of patients who present with pericardial effusion in the setting of acute pericarditis will complain of pleuritic (pericarditic)-type chest pain [36]. The rate of accumulation of pericardial fluid is the main parameter correlating with symptoms’ development, since slowly accumulating effusion may be asymptomatic even if large [6]. In contrast, small but fast-accumulating effusions, which may be observed after cardiac trauma, may cause significant hemodynamic impairment. 

Clinical examination in large pericardial effusions may reveal muffled heart sounds, jugular vein distension, and occasionally peripheral stasis [6]. Pulsus paradoxus during blood pressure measurement is the hallmark for the diagnosis of cardiac tamponade [5]. 

Electrocardiogram depicts the characteristic first-phase findings of diffuse concave ST-segment elevations and PR-segment depression in the absence of q waves in approximately 50% of patients with acute pericarditis and pericardial effusions [6,36]. Low-voltage and electrical alternans may appear in large pericardial effusions with swinging heart [6]. Chest X-ray should be performed in all cases according to the relevant 2015 European Society of Cardiology Guidelines; however, its diagnostic accuracy is low since enlargement of cardiac silhouette develops in the presence of at least moderate effusions with the accumulation of more than 250–300 mL of fluid [5]. In large effusions, chest X-ray depicts the characteristic water bottle configuration in the absence of pulmonary congestion [5].

Blood work should include routine tests with the addition of C-reactive protein, thyroid-stimulating hormone, and troponin. Further tests will be performed on an individualized basis according to the specific clinical scenario [6].

Echocardiography is the mainstay for the assessment of patients with pericardial syndromes, including pericardial effusions [6]. As already mentioned, echocardiography allows for the quantification of pericardial effusion, but most importantly, the hemodynamic impact of pericardial effusion to the diastolic heart function and overall performance [34]. Echocardiography has the advantage of the possibility of being performed at the bed side without the adoption of radiation and has an ideal profile for the follow-up of those patients in terms of safety, cost, and feasibility [34]. Echocardiography may detect early signs heralding cardiac tamponade with a high accuracy, allowing for a timely intervention for the prevention of this catastrophic complication. A summary of the main echocardiographic findings observed in near or overt cardiac tamponade, along with their sensitivity and specificity, is depicted in Table 2 [5,6,34].

To sum up the medical history, clinical examination with emphasis on heart auscultation, chest X-ray, electrocardiography, echocardiography, and routine blood tests should be performed in all cases of pericardial effusions and pericardial syndromes overall [6]. However, since the etiology, as already mentioned, remains occult in about half of pericardial effusion cases, the adoption of a second level of investigations is occasionally needed, including computed tomography, cardiac magnetic resonance imaging, pericardial drainage with pericardial fluid analysis (general chemistry, cytology, biomarkers, polymerase chain reaction, and microbiology), pericardioscopy with pericardial biopsy, mammography, positron emission tomography/computed tomography, and further blood tests such as the *QuantiFERON*-TB Gold test and serology for autoimmune–autoinflammatory disorders, among others [5,6,34]. It should be emphasized that the traditional Light’s criteria employed for the characterization of pleural effusions according to recent evidence do not apply for pericardial fluid [37]. Specifically, due to the high content of pericardial fluid in mesothelial cells along with the high concentration of proteins, albumin, and lactate dehydrogenase (values which are notably higher in pericardial fluid than in the blood), pericardial fluid may be erroneously classified as exudate [37]. Specific criteria for the classification of pericardial fluids are currently under investigation. Moreover, clinicians should be aware of the temporary elevation of C-reactive protein after pericardiocentesis due to pericardial irritation and tube placement. This is important in terms of the characterization of pericardial effusion as inflammatory or not. To avoid misinterpretations, plasma C-reactive protein values should be always measured before the procedure [38].

Computed tomography has the advantage of uncovering comorbid conditions accounting for pericardial effusions and providing anatomical details on localized effusions not easily assessed by echocardiography. Moreover, with the adoption of attenuation values (Hounsfield units), valuable information on the composition of pericardial fluid may be obtained [5]. In particular, values between −60 and −80 characterize chylopericardium, <10 U are suggestive of transudative effusion, those between 10 and 60 characterize purulent, malignant, or myxomatous effusions, while values >60 U are compatible with hemorrhagic effusions [5,39]. Cardiac magnetic resonance imaging is the only imaging procedure with the ability of tissue characterization [6]. In recent years, its use has been increasingly expanding for the investigation of pericardial syndromes, including pericardial effusions [24]. With the adoption of the T2-weighted short-tau inversion recovery technique, the presence of pericardial edema compatible with acute pericardial inflammation has been detected [24]. On the other hand, late gadolinium enhancement by conventional phase-sensitive inversion recovery is found in both the acute and subacute phase of pericardial inflammation. In contrast, the value of the T1 and T2 mapping quantitative assessment in identifying pericardial inflammation is less studied [24]. 

## 5. Management of Pericardial Effusions

The current European Society of Cardiology Guidelines on pericardial diseases include a very elegant and operational algorithm for the management of patients with pericardial effusions. The algorithm consists of four consecutive steps [6]. Progression to a subsequent step is performed if the condition described in the previous step is not fulfilled by the individual patient. 

According to the latter algorithm, the patient should be investigated for the presence of cardiac tamponade, neoplastic pericarditis, and evidence of purulent effusion as a first step. Cardiac tamponade is a life-threatening condition which should be promptly recognized and treated [40]. In the presence of clinical suspicion, physical examination, including blood pressure measurement and point of care echocardiography, is sufficient for diagnosis [5]. It should be stressed that in viral/idiopathic pericarditis, which is the most common cause of pericarditis in Western countries, the development of cardiac tamponade is rare (~1.2% of cases), but it is quite common in specific/secondary forms of acute pericarditis (up to ~20%) [41]. Patients with cardiac tamponade should undergo pericardial drainage by any technique (pericardiocentesis or pericardial window) according to local expertise [6]. Pericardiocentesis should be performed under echocardiographic or fluoroscopic guidance to minimize the possibility of procedure-related complications [6,42]. In unfortunate patients with cardiogenic shock in the context of cardiac tamponade, cardiorespiratory resuscitation will fail if drainage is not promptly performed [5,43]. In selected cases, in centers without cardiac catheterization laboratory or inability to deal with such cases, pericardiocentesis in patients with near-tamponade features may be postponed for 48 h, allowing the patient to be transferred in a specialized center [44]. In this context, a three-step scoring system has been proposed by the European Society of Cardiology Working Group on Myocardial and Pericardial Disease to make a decision on the timing of pericardiocentesis [44]. This score system includes a variety of etiological, clinical, and imaging parameters. Although it lacks validation, it is a useful tool for clinical decision making. Specifically, if the cumulative score is >6, then emergent pericardiocentesis should be performed in situ. For lower scores, pericardiocentesis may be deferred, allowing the patient to be transferred to a center with sufficient expertise in pericardial drainage [44].

Another important issue to take into account when pericardiocentesis is performed is the avoidance of the rapid evacuation of the pericardial space. The latter approach has been associated with a life-threatening complication, which is pericardial decompression syndrome [45]. The abovementioned syndrome is observed in ~5% of cases within 48 h from pericardial drainage [5,46]. Possible clinical manifestations include cardiogenic shock in ~70% of cases and pulmonary edema in the rest. Treatment is supportive, the reported mortality rate is as high as 30%, and rapid evacuation (>1 L) is considered an important risk factor [5,44]. As a rule, gradual and prolonged decompression until pericardial fluid return ~20–30 mL/day is proposed as the most appropriate drainage technique in order to avoid complications and prevent pericardial effusion recurrences [47,48].

The second condition where pericardial drainage is proposed, if technically feasible, is the suspicion of neoplastic pericarditis. Patients with a known malignancy and especially those with lung and/or breast cancer, as well as those with hematologic malignances, are mainly at risk of malignant pericarditis [6,49,50]. Cardiac tamponade with hemorrhagic effusion along with normal or near-normal plasma C-reactive protein values are red flags for the diagnosis of neoplastic pericarditis [18]. Cytological analysis of the pericardial fluid will confirm or exclude this possibility [34]. Apart from diagnostic purposes, the identification of malignant cells in the pericardial fluid is of paramount importance in terms of outcome, since those patients have an ominous prognosis [51]. Samples with a minimal volume of at least 60 mL should be sent as soon as possible for cytological analysis in order to increase the diagnostic yield of the procedure [52]. Cytology along with multimodality imaging have contributed to an improved identification of patients with neoplastic pericarditis, which is important in terms of risk stratification and treatment decisions [5].

Finally, the third clinical scenario requiring pericardiocentesis according to the relevant 2015 European Society of Cardiology Guidelines recommendations is the suspicion of purulent pericarditis [6]. This entity is more often, but not exclusively, observed in immunocompromised patients, with more unusual microorganisms often being involved [6,53]. Clinical manifestations include high fever and rapid progression to septic shock if untreated [6]. Prolonged antibiotic therapy, pericardial window with debridement of the pericardial cavity, or the intrapericardial administration of saline (with or without intrapericardial fibrinolysis) may expediate recovery and lower the rate of progression to constrictive pericarditis in the medium term. 

**Figure 1:**
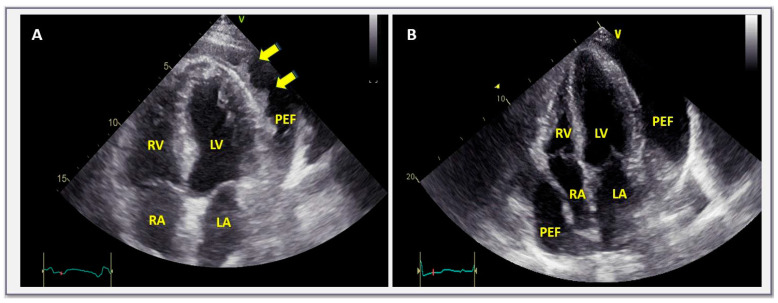
Transthoracic echocardiographic apical 4-chamber view in a case with acute pericarditis with pericardial effusion (**A**) and a large, chronic, non-inflammatory, idiopathic, pericardial effusion (**B**). In (**A**), fibrin strands due to ongoing pericardial inflammation (C-reactive protein was ~200 mg/L) are observed within the pericardial space. In contrast, in (**B**), pericardial space is filled with clear liquid (C-reactive protein in this case was ~1 mg/L—normal values < 5). LV = left ventricle, RV = right ventricle, LA = left atrium, RA = right atrium, PEF = pericardial effusion.

When none of the three aforementioned conditions are fulfilled, then a C-reactive protein measurement should be performed as a second step approach according to the pertinent European Society of Cardiology algorithm [6]. In the presence of elevated C-reactive protein elevation, pericardial effusion should be perceived as of inflammatory origin and empiric anti-inflammatory treatment including colchicine should be offered to the patient (Figure 1) [6]. Since C-reactive protein is a non-specific marker of inflammation, in doubtful cases or in those with marginal C-reactive protein elevation, cardiac magnetic resonance imaging will contribute to establishing a definite diagnosis [5,6,24].

In the absence of inflammatory markers’ elevation, investigation for a secondary condition known to be associated with pericardial effusions (e.g., systemic autoimmune/autoinflammatory diseases) should be undertaken (third step of the algorithm) [6]. At present, multimodality imaging has consistently enhanced the identification of possible diagnoses. Patients with large effusions in general have a greater possibility of being diagnosed with specific conditions in ~60% of cases [54]. The multidisciplinary approach of patients with pericardial effusion in the setting of a specific etiology is of paramount importance for an optimal treatment strategy tailored to the individual’s etiology [5]. 

Finally, if the previously reported conditions are excluded upon extensive work-up, as a last (and fourth) step, the size of the pericardial effusion should be taken into consideration [6]. In the presence of small or moderate effusions, regular follow-up every 3–6 months is sufficient. In contrast, in cases of chronic (lasting more than 3 months) large pericardial effusions, pericardial drainage should be considered according to the Guidelines [6].

## 6. Asymptomatic, Large, Idiopathic, Non-Inflammatory Effusions in Light of Current Evidence

Approximately 10 years after the publication of the latest European Society of Cardiology Guidelines on Pericardial Diseases, ongoing research has provided new evidence on pericardial syndromes. As a result, the European Society of Cardiology has already anticipated the release of new Guidelines on pericardial diseases and myocarditis in a single document, due in the summer of 2025. In relation to the topic of this review, recent investigations published after the 2015 Guidelines have challenged the practice of performing pericardial drainage in asymptomatic subjects, with large, chronic, idiopathic, C-reactive-protein-negative pericardial effusions [8,9,10].

Historically, this recommendation was mainly based on an earlier study on 28 patients, with the latter clinical profile showing the development of cardiac tamponade in 30% of cases over a follow-up period of 7 years [7].

Several years later, in a larger prospective cohort study enrolling 100 patients followed-up for a mean of 50 months (mean age 61.3 years, 44 men), the same topic has been revisited [8]. The first important observation in this study was that 44% of patients were asymptomatic at presentation. The second clue was that the rate of tamponade during follow-up was fairly lower than previously described, namely 8% overall or 2.2% per year. Last and most important, the mortality rate during follow-up was 0%, which is due to the advice given to the patients to seek prompt medical care in the case that symptoms compatible with tamponade should appear. Notably, in 40% of cases, pericardial effusion regressed during follow-up. On the other hand, 42% of patients required pericardial drainage during the study period, and this patient subgroup depicted a significantly worse recurrence-free survival and complications-free survival in comparison to the conservatively treated subgroup. Thus, this study showed for the first time that routine drainage is not beneficial in patients with this clinically profile, especially if asymptomatic [8].

**Figure 2:**
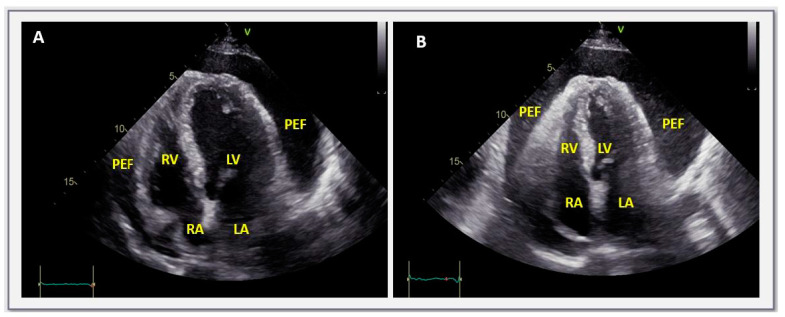
Transthoracic echocardiographic apical 4-chamber view in a case with asymptomatic, large, chronic, non-inflammatory (C-reactive protein was ~1 mg/L, normal values < 5), idiopathic, pericardial effusion. (**B**) Obtained ~15 months after (**A**). Notably, the size of pericardial effusion was virtually unchanged, with the patient still being asymptomatic, without restrictions in physical activity. LV = left ventricle, RV = right ventricle, LA = left atrium, RA = right atrium, PEF = pericardial effusion.

Further evidence on these challenging patients was offered by a second study which was similar in terms of clinical background to the previous one, with a difference, however, of including exclusively asymptomatic individuals [9]. Among the 74 subjects enrolled, based on the attending clinician’s advice and personal preferences, 39 underwent pericardiocentesis, 13 underwent pericardial window, and 22 were treated conservatively. The median follow-up was 24 months. The main finding of this study was that pericardiocentesis did not offer a long-lasting result since effusion re-accumulation was observed in the majority of cases (~77% overall and similar to the baseline amount in 41%). The longer the duration of the volume of the effusion, the greater the probability of re-accumulation. The relevant rates of fluid re-accumulation after pericardial window were 15.4% and 7.7%, respectively. Notably, pericardial drainage by any means was accompanied by a non-negligible rate of procedure-related complications (up to 15%) without, however, deaths. On the other hand, conservatively treated patients developed symptoms and required drainage in ~9% of cases, while the effusion regressed (partially or completely) in 14% of cases and persisted with a similar size in the remainder of cases (77%) (Figure 2) [9].

Finally, additional clues on the ongoing debate regarding the optimal management of large pericardial effusions in asymptomatic or oligosymptomatic patients were obtained by a multicenter international retrospective study including 124 patients (mean age 64 years, 50% female) which were followed-up for 29.6 ± 25.6 months [10]. Most of these study patients (namely 89%) underwent pericardiocentesis, and the remainder underwent pleuro-pericardial window. In line with the previous study, pericardial fluid re-accumulation was observed in 61.8% of cases. Interestingly, ~17% of patients developed post-pericardiotomy syndrome within 6 weeks after pericardial drainage [10].

To summarize, the results of the aforementioned investigations suggest the need for caution regarding the use of pericardial drainage in chronic and large oligo- or asymptomatic patients and C-reactive-protein-negative pericardial effusions. In view of the new evidence, this condition has an overall benign course, and a non-invasive watchful waiting approach is the most appropriate choice in clinical practice. Based on the accumulated recent data, the 2015 Guidelines recommendation for this patient subgroup should eventually be reconsidered. 

An issue of concern regarding pericardial drainage by any means in asymptomatic patients is that every procedure may eventually be accompanied by complications. In the context of pericardiocentesis, depending on the pericardiocentesis access site (namely subxiphoid, parasternal, or apical), possible periprocedural complications most commonly include right heart chambers or left ventricular puncture, pneumothorax, puncture of the internal thoracic vessels and coronary arteries, as well as left liver lobe puncture [5]. Notably, the abovementioned complications may be catastrophic if not promptly recognized and adequately treated. Additional short/medium-term complications related to pericardiocentesis consist of bacterial pericarditis and, as already mentioned, post-cardiac injury syndrome as well as pericardial decompression syndrome [5,10,55]. The reported rate of complications after pericardiocentesis varies widely in the literature depending on the specific clinical scenario such as population studied, underlying disease, and emergent or scheduled setting of the procedure. Major complications have been reported in ~4% of cases, with the relevant rate depending on the operator’s expertise [5]. Procedure guidance by ultrasound of fluoroscopy is of paramount importance for minimizing the risk of complications. 

Surgical pericardial window, on the other hand, is a safe and minimally invasive procedure which allows for the visualization of pericardium and adjacent structures in contrast with pericardiocentesis, which is a ‘blind’ procedure. The optimal method for pericardial window creation is a matter of controversy. Possible options include the creation of pericardial window through video-assisted thoracoscopic surgery (VATS), which has the advantage of being a minimally invasive technique or the formation of a pleuro-pericardial window through the anterior parasternal approach [56]. Both approaches are safe and efficacious, with local expertise being the most important parameter for the selection of the procedure. Subxiphoid window should preferably be avoided since communication with the pleural cavity cannot be accomplished with this technique. Risks related to pericardial window creation are related to general anesthesia, while additional complications include bleeding, infections, post-cardiac injury syndrome, pneumomediastinum, etc. [9]. 

## 7. Prognosis

As it has been emphasized throughout this review, the most important parameter affecting prognosis in pericardial effusions is etiology [6]. In general, small and idiopathic pericardial effusions have an excellent prognosis, although not all studies concur with this statement [6,17]. On the other hand, malignant pericardial effusions have a dreadful outcome [57]. Thus, it is of paramount importance in terms of risk stratification to perform an accurate etiology search in patients with pericardial effusions, especially in those moderate or large, in order to establish a diagnosis and administer a tailored treatment to the patient [6]. 

According to a metanalysis’s findings, the detection of pericardial effusions in diseases known to be potentially associated with such effusions (e.g., heart failure, pulmonary arterial hypertension, and malignancy, among others) is a marker of disease severity [58].

**Figure 3:**
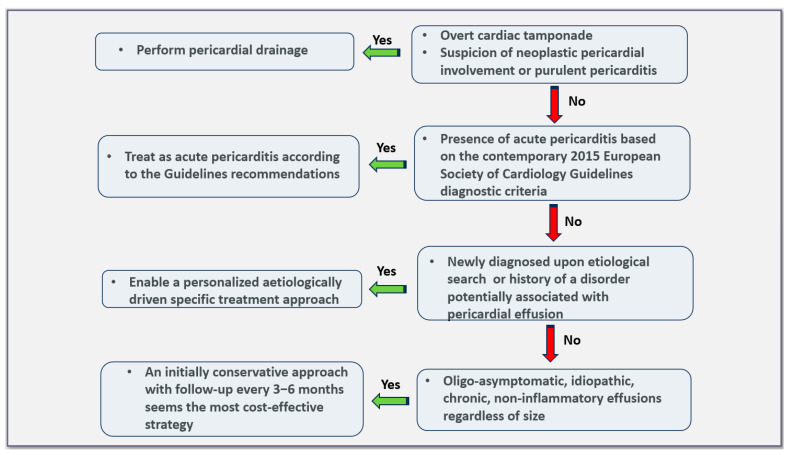
Proposed triage and management of pericardial effusion based on the most recent evidence.

As already mentioned, the clinical course of moderate and large idiopathic and non-inflammatory effusions, in the absence of symptoms, is mostly benign, and routine drainage is discouraged [8,9,10,59]. Nevertheless, regular follow-up (clinical and echocardiographic) should be performed every 3 to 6 months, along with the advice of seeking timely medical care, should changes in the clinical status appear (Figure 3) [6].

## 8. Specific Considerations—Gaps in Knowledge

Despite the overall benign course of chronic, asymptomatic, idiopathic, non-inflammatory effusions, there are some areas where the clinical decision making is challenging and controversial. It should be emphasized that independently of the individual clinical scenario, all patients with pericardial effusions should be advised to promptly seek medical attention in cases of symptoms onset to exclude an increase in the size of the effusion [5]. In the same line, in the case of the appearance of symptoms compatible with acute pericarditis, these patients should seek medical advice without delay. In the latter cases, if pericarditis is confirmed, the production of additional fluid may account for the development of cardiac tamponade. Thus, in similar cases, hospital admission, close monitoring, and aggressive treatment are required, along with vigilance for prompt pericardial drainage in the case of hemodynamic deterioration. 

Additional clinical scenarios possibly requiring a more aggressive approach encompass among other individuals with moderate–large effusions that do not have easy access to health care services. Likewise, for instance, mariners employed in long-lasting transoceanic travels or other workers with similar professional obligations are problematic groups of patients. 

A particular word of caution applies to cases of newly, incidentally diagnosed pericardial effusions, even if asymptomatic and non-inflammatory. These patients should be closely followed and checked for effusion stability (for instance, every 2 weeks) for 3 months, a time which is sufficient for the diagnostic work-up to be completed and for the effusion to be labeled as chronic [5,24].

Three additional groups of challenging patients encompass young women planning pregnancy and athletes. It is well known that in normal pregnancy, approximately 40% of women develop usually mild and less frequently moderate pericardial effusion in the absence of pericarditis [60]. Thus, a woman with a known pericardial effusion should be aware of this possibility before childbearing and be treated accordingly upon shared decision-making in a multidisciplinary context [61].

On the other hand, data on exercise safety in cases with pericardial effusion are lacking. The 2020 European Society of Cardiology Guidelines on Sports Cardiology and Exercise in patients with cardiovascular disease does not recommend exercise restriction and allows participation in sports in asymptomatic individuals with small, idiopathic, pericardial effusions incidentally found during a routine echocardiographic study [62]. However, no specific recommendations have been given for asymptomatic athletes with moderate and large effusions. In these cases, whilst waiting for solid data, decisions should be taken in an individualized manner. Ergospirometry is a useful test used in decision making in similar cases [5]. 

A third condition where pericardial effusions are commonly found is pectus excavatum. The latter structural deformity is encountered in 0.5% in the general population (0.6% in women and 0.4% in men) [63]. Pericardial effusion is observed in one third of patients with this condition, and in ~10% of cases, the effusion is moderate (>10 mm). Pericardial events in this setting are rare and in the absence of symptoms, regular follow-up is recommended [63].

Last but not least, asymptomatic patients with at least moderate effusions should be flowed up in specialized pericardial units to ensure an optimal follow-up and clinical guiding in this debated condition. Patients should be reassured about the benign course of this condition in most cases and instructed to seek prompt medical advice in cases of symptoms onset or new clinical features suggestive of a specific condition, such as an occult autoimmune disease [64]. Patient preference should be taken into account upon detailed explanation of the pros and cons of conservative or invasive strategy. In the latter case, it should be stressed that pericardiocentesis is a less invasive procedure but with poor medium-term results in terms of re-accumulation. In contrast, pleuro-pericardial window, ideally with video-assisted thoracoscopic surgery, is the most durable option and, at the same time, a minimally invasive surgical technique. 

## 9. Conclusions 

Chronic pericardial effusion is a problematic and embarrassing pericardial syndrome that has been subject to considerable controversy for many years due to a lack of evidence-based data on its appropriate management. However, ongoing clinical research has contributed to a better understanding and management of this condition. According to the new evidence, the recommendation of routine drainage should be reconsidered in view of the benign overall course of the disease and the non-negligible rate of complications (including a considerable rate of acute pericarditis due to pericardiocentesis-related post-cardiac injury syndrome) associated with the procedure. The forthcoming 2025 Guidelines are eagerly awaited to provide updated and specific recommendations on this troublesome entity. 

## Figures and Tables

**Table 1:** Classification of pericardial effusions.

Size:	Small: <10 mm
	Moderate: Between 10 and 20 mm
	Large: >10 mm
Onset:	Acute: <1 week
	Subacute: Between 1 week and 3 months
	Chronic: >3 months
Distribution:	Circumferential
	Localized
Composition:	Transudate
	Exudate inflammatory effusions, hemopericardium, pyopericardium, chylopericardium, pneumopericardium
Hemodynamic effects:	Hemodinamically insignificant
	Cardiac tamponade
	Effusive-constrictive

**Table 2:** Main echocardiographic findings with relevant sensitivities and specificities of near (imminent) or overt cardiac tamponade in patients with pericardial effusions (findings are reported with respect to decreasing sensitivity).

Variable	Specificity	Sensitivity
Right atrial collapse (inversion) with duration of atrial collapse to cardiac cycle duration >0.34	100%	>90%
Right ventricular collapse	72–100%	48–100%
Inferior vena cava enlargement (>20 mm) with blunted respiratory response (<50% with inspiration)	40%	97%
Swinging heart	N.A.	N.A.
Respiratory variation of 25% or more in transmitral early diastolic filling (E) velocity	N.A.	N.A.
